# Effect of temperature on the orthodontic clinical applications of niti closed-coil springs

**DOI:** 10.4317/medoral.19073

**Published:** 2013-05-31

**Authors:** Eduardo Espinar-Escalona, José M. Llamas-Carreras, José M. Barrera-Mora, Camilo Abalos-Lasbrucci, Francisco J. Gil-Mur

**Affiliations:** 1 Grupo de Ortodoncia. Facultad de Odontología. Universidad de Sevilla; 2 Dept. C.Materiales e Ingeniería Metalúrgica. ETSEIB. Universidad Politécnica de Cataluña. Barcelona. Spain

## Abstract

NiTi spring coils were used to obtain large deformation under a constant force. The device consists on a NiTi coil spring, superelastic at body temperature, in order to have a stress plateau during the austenitic retransformation during the unloading. The temperature variations induced changes in the spring force. Objectives: The aim of this study is to investigate the effect of the temperature variations in the spring forces and corrosion behaviour simulating the ingestion hot/cold drinks and food. Study Design: The springs were subjected to a tensile force using universal testing machine MTS-Adamel (100 N load cell). All tests were performed in artificial saliva maintained at different temperatures. The corrosion tests were performed according to the ISO-standard 10993-15:2000. Results: The increase in temperature of 18oC induced an increase in the spring force of 30%. However, when the temperature returns to 37oC the distraction force recovers near the initial level. After cooling down the spring to 15oC, the force decreased by 46%. This investigation show as the temperature increase, the corrosion potential shifts towards negative values and the corrosion density is rising. Conclusions: The changes of the temperatures do not modify the superelastic behaviour of the NiTi closed-coil springs. The corrosion potential of NiTi in artificial saliva is decreasing by the rise of the temperatures.

** Key words:**Superelasticity, NiTi, springs, orthodontic, coils, recovery, temperature.

## Introduction

NiTi shape memory alloys coil springs have been a useful intraoral tool for space closure and opening in different orthodontic therapies. Open-coil springs are designed to deliver an expansion force as indicated in space opening (different movement ofteeth), whereas the closed-coil springs are intended to deliver, compressive forces (ex. retraction of the canines). These NiTi springs produce continuous, light forces over range of activation. Conventional coil springs (stainless steel, titanium) display a linear plot in the force deflection graph; whereas superelastic NiTi springs show a typical force plateau. The stress-strain curve for a superelastic material shows that the stress which induced the phase transformation (martensite-parent) upon unloading takes place at a fairly constant stress over a significant range of wire activation. These low and continuous loads are adequate to bio-logical response (1-3).

It is generally assumed that the optimal tooth movement is achieved by applying forces that are low in magnitude and continuous in nature. Such forces minimize tissue destruction and produce a relatively constant stress in the periodontal ligament during tooth movement (4). The superelasticity of NiTi archwires allows the orthodontist to apply an almost continuous light force with larger activations that results in the reduction of tissue trauma and the patient discomfort, thus facilitating enhanced tooth movement (5).

Oral cavity is one of the most inhospitable environments in the human body. Therefore, orthodontic archwires, coils or brackets are subject to larger temperature variations than most other parts, coping with ice cold temperatures (5ºC) through to hot coffee and soup (70ºC). Many works about effect of different parameters on the corrosion behaviour of NiTi orthodontic wires have been published (6,7) but few have rarely investigated the effect of temperature on the corrosion behaviour of NiTi alloys by electrochemical techniques in artificial saliva.

NiTi alloys combine their shape memory effect and superelasticity with excellent mechanical properties and a good biocompatibilty (3,8). Despite of these advantages, it is necessary to study the effect of the variations of temperature which can produce important changes in the load applied to the teeth and the corrosion behaviour. These temperature variations in the spring forces simulate the ingestion hot/cold drinks and food.

## Material and Methods

The device is simple and consists of a NiTi spring that is commercially available for orthodontic applications. GAC-Orthospain consists of 52% at nickel and 48% at titanium.

Fifty NiTi closed 300 g coil springs were obtained by GAC-Orthospain (reference 10-000-20). The springs have an external diameter of 1.1 mm, a wire diameter of 0.3 mm, a working length of 2.7 mm (9 coils), and a theoretical discharge force of 300 gf (grames/force) (this is the force in which the unloading plateau should occur).

The springs were subjected to a tensile force using universal testing machine MTS-Adamel (100 N load cell). All tests were per-formed in artificial saliva maintained at different temperatures. The chemical composition of the artificial saliva is shown in  Table 1. The following tensile test were performed: A tensile force was applied at a strain rate of 2 mm/min up to 8 mm (3.9 times the original length) followed by a complete unloading at 1 mm/min.

**Table 1:**
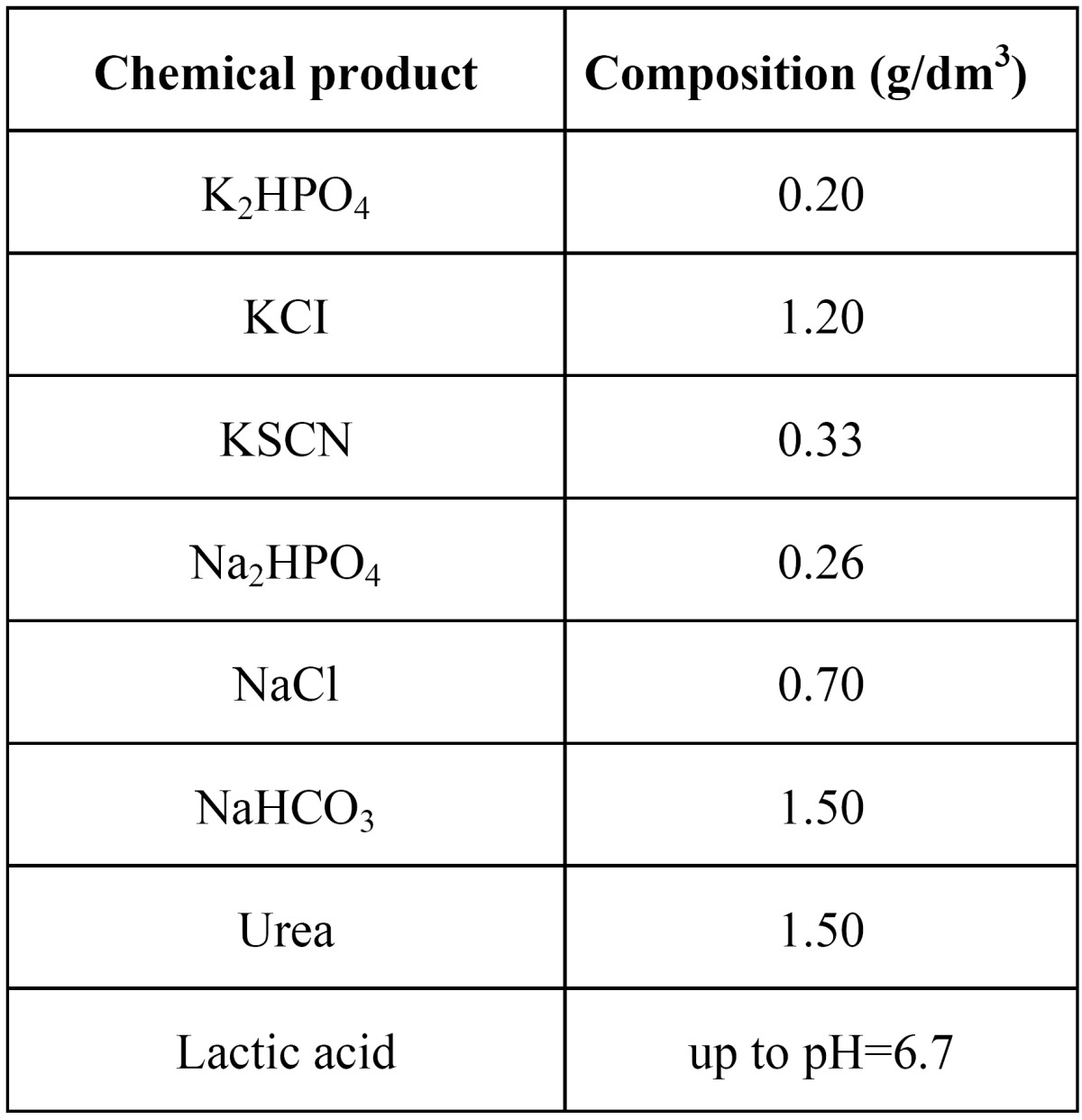
Chemical composition of the artificial saliva.

**Figure 1:**
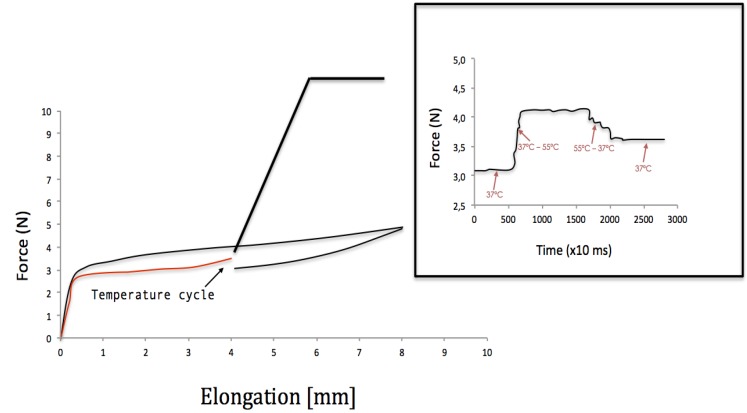
Mechanical test simulating a hot ingestion of food/beverage.

To simulate the change of temperature when ingesting food or drinks, the bath temperature was changed during the unloading period. Springs were first loaded at 2 mm/min and unloaded at 1 mm/min. However, during unloading the displacement was stopped and fixed at 4 mm. At this position, the temperature of the bath was changed to either 55oC or 15oC to simulate respec-tively the ingestion of a hot or cold beverage (9). Subsequently, the temperature was returned to 37oC, and the unloading of the spring continued (Fig. 1).

Closed-coil springs were sectioned in order to produce corrosion testing samples. A 25 mm of each coil spring was cut with a sterile orthodontic plier and was isolated with wax at the point of interphase between the testing solution and the air. The corrosion tests were performed according to the ISO-standard 10993-15:2000 “Biological evaluation of medical devices. Part 15: Identification and quantification of degradation products from metals and alloys”; this standard is commonly used in orthodontic archwires corrosion (6-11).

The tests were carried out with a Voltalab PGZ 301 potentiostat (Radiometer, Copenhagen, Denmark) controlled by Voltamaster 4 software (Radiometer Analytical, Villeurbanne Cedex, France). The testing solution was artificial saliva that was kept at adifferent temperatures. The reference electrode was an Ag/AgCl/KCl electrode (Eº=0.222V). The auxiliary electrode used was a Platinum electrode and had a surface of 240 mm2 (Radiometer Analytical, Villeurbanne, France).

The Open Circuit Potential was monitored for 3 hours in order to allow a leveling off of the value before the polarization resistance test. The CV assay was performed by scanning the potential of the alloy of the sample at 0.25 mV/s with the minimum current set at -1 A and the maximum at +1 A with a minimum range set at 100 ?A between -300 mV and +2000 mV (the upper limit for TMA archwires was +2700 mV) around the OCP value. Corrosion potentials (Ecorr) and corrosion currents (icorr) were recorded for the different samples tested.

## Results

The increase in temperature (Fig. 1) of 18oC induced an increase in the spring force of 30%. However, when the temperature returns to 37oC the distraction force recovers near the initial level. After cooling down the spring to 15oC, the force decreased by 46%. As observed for an increase of temperature, the spring force returns to the original value when the temperature goes back to 37oC. In both cases it is important to note that, although the force does not return exactly to the original level when the temperature is again 37oC, it does when the distraction process continues. This test was repeated 10 times and the results were the same. The number of cycles did not modify the force-displacement curve.

The corrosion current densities were obtained from the polarization curves by Tafel using cathodic and anodic slopes of the polarization curves. The electrochemical parameters are shown in  Table 2. These results show as the temperature increase, the corrosion potential shifts towards negative values and the corrosion density is rising.

**Table 2:**
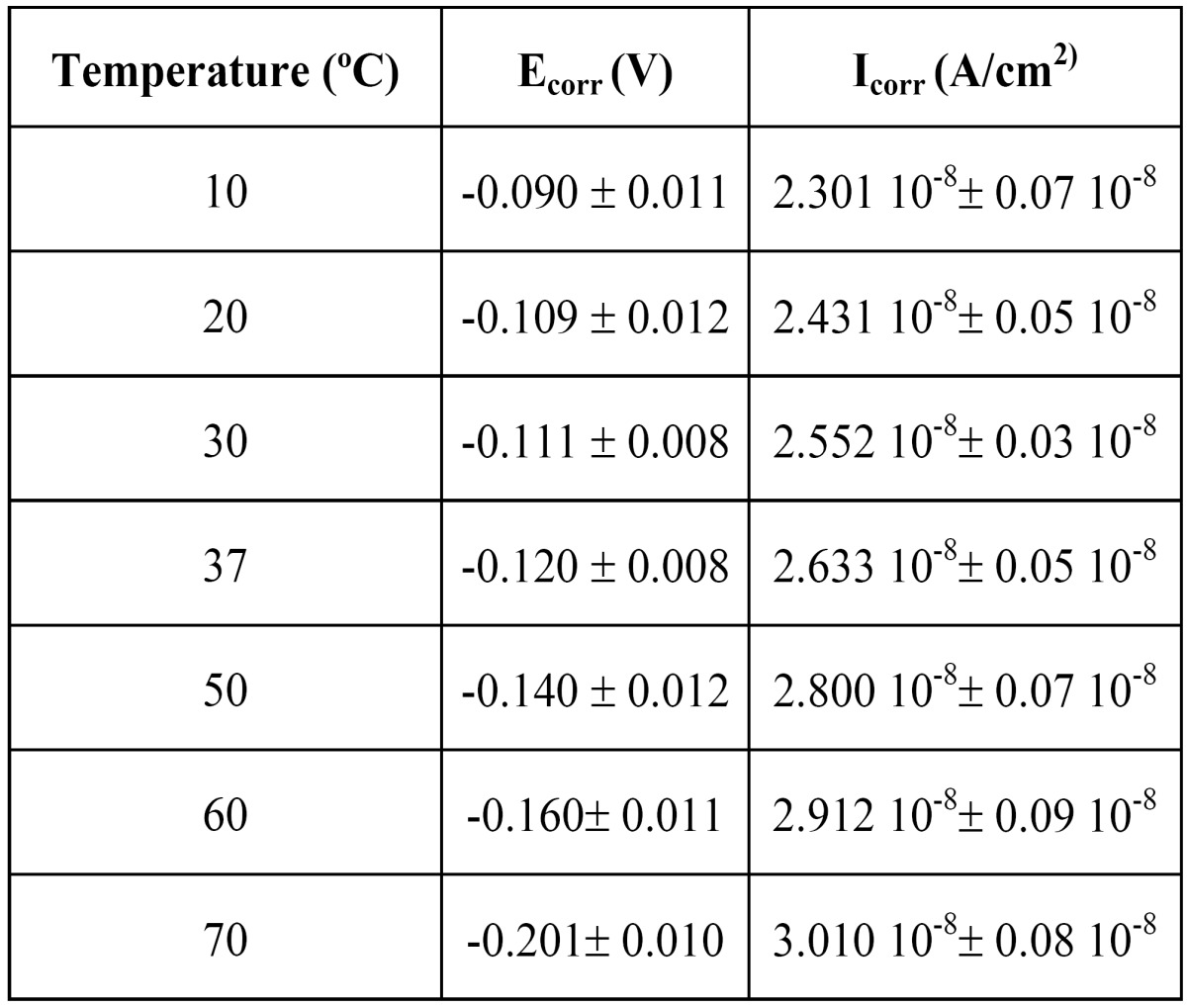
Corrosion parameters of NiTi coil spring at different temperatures.

## Discussion

An important parameter that was investigated in this study is the variation of temperature during distraction. When the temperature varies, the force applied by the device changes. When heating, the martensite transforms back to austenite. However, in contrary to what has been reported by Suarez (2) for flexion forces on orthodontic wires, the force returns near the original level when the temperature returns to 37º C. This indicates a retransformation austenite - force induced martensite. The cooling case is simpler; there is a higher percentage of martensite and these results in a force decrease. However, this phenomenon is reversible when heating again. It is not clear though whether this increment on tissue stresses due to an increase of temperature can damage the tissues. In such a case the patient nutrition should be controlled to avoid warm food or beverage. If the stress increase does not damage the tissues, the teeth movement will continue as normal since the spring force returns to its original plateau.

The corrosion current density is directly proportional to corrosion rate, the higher values of current density is an indication of lower resistance against corrosion (9-13). The low corrosion current densities (about 108) produce an improvement of corrosion resistance, even at higher temperatures than oral environment (37ºC) (14-17). This fact is due to the formation of a protective oxide film constituted by titanium oxide (3,17-21).

The changes of the temperatures do not modify the superelastic behaviour of the NiTi closed-coil springs. The corrosion potential of NiTi in artificial saliva is decreasing by the rise of the temperatures.
